# Perioperative Management of Pheochromocytoma: From a Dogmatic to a Tailored Approach

**DOI:** 10.3390/jcm10163759

**Published:** 2021-08-23

**Authors:** Salvatore Buscemi, Giuseppe Di Buono, Rocco D’Andrea, Claudio Ricci, Laura Alberici, Lorenzo Querci, Saverio Selva, Francesco Minni, Roberto Citarrella, Giorgio Romano, Antonino Agrusa

**Affiliations:** 1 Department of Surgical, Oncological and Oral Sciences (Di.Chir.On.S.), University of Palermo, 90127 Palermo, Italy; salvatore.buscemi02@unipa.it (S.B.); giuseppe.dibuono@unipa.it (G.D.B.); giorgio.romano@unipa.it (G.R.); antonino.agrusa@unipa.it (A.A.); 2 Division of Anaestesioloigist, IRCCS Azienda Ospedaliero-Universitaria Di Bologna, Via Albertoni 15-Italia, 40121 Bologna, Italy; rocco.dandrea@aosp.bo.it; 3 Division of Pancreatic Surgery, IRCCS Azienda Ospedaliero-Universitaria Di Bologna, Via Albertoni 15-Italia, 40121 Bologna, Italy; lau.alberici@gmail.com (L.A.); saverio.selva@aosp.bo.it (S.S.); francesco.minni@unibo.it (F.M.); 4 Department of Internal Medicine and Surgery (DIMEC), Alma Mater Studiorum, University of Bologna, 40121 Bologna, Italy; querci.lorenzo@gmail.com; 5 Department of Health Promotion Sciences Maternal and Infantile Care, Internal Medicine and Medical Specialties (PROMISE), University of Palermo, 90127 Palermo, Italy; roberto.citarrella@unipa.it

**Keywords:** pheochromocytoma, alfa-blockers, perioperative management

## Abstract

Background: Perioperative management of pheochromocytoma (PCC) remains under debate. Methods: A bicentric retrospective study was conducted, including all patients who underwent laparoscopic adrenalectomy for PCC from 2000 to 2017. Patients were divided into two groups: Group 1 treated with alpha-blockade, and Group 2, without alfa-blockers. The primary end point was the major complication rate. The secondary end points were: the need for advanced intra-operative hemostasis, the admission to the intensive care unit (ICU), the length of stay (LOS), systolic (SBP), and diastolic blood pressure (DBP). Univariate and multivariate analysis was conducted. A *p*-value < 0.05 was considered statistically significant. Results: Major postoperative complications were similar (*p* = 0.49). Advanced hemostatic agents were 44.9% in Group 1 and 100% in Group 2 (*p* < 0.001). In Group 2, no patients were admitted to the ICU, while only 73.5% of Group 1 (*p* < 0.001) were admitted. The median length of stay was larger in Group 1 than in Group 2 (*p* = 0.026). At the induction, SBP was 130 mmHg in Group 1, and 115 mmHg (*p* < 0.001). The pre-surgery treatment was the only almost statistically significant variable at the multivariate analysis of DBP at the end of surgery. Conclusion: The preoperative use of alfa-blockers should be considered not a dogma in PCC.

## 1. Introduction

Pheochromocytoma (PCC) is a neuroendocrine tumor originating from the chromaffin cell in the adrenal medulla that secretes the catecholamine. It has a low incidence of 0.8 per 100,000 individuals/year, occurring in 0.1–0.6% in the hypertensive population [1,2].

The secreted catecholamines explain all the clinical manifestations of this type of tumor. Because the release of catecholamine by PCC is unpredictable, the clinical presentation includes headaches, sweating, tachycardia, palpitation, and hypertension and can range from clinically asymptomatic to life-threatening hypertensive crises [3].

To date, surgical resection is the only cure. Although laparoscopic resection was once considered not indicated in PCC, several studies in the last twenty years have demonstrated that laparoscopic adrenalectomy is associated with less pain, less morbidity, and quick recovery. It is considered the gold standard compared to open adrenalectomy [4,5,6].

However, general anesthesia induction, abdominal pressure fluctuations, and direct tumor handing could increase catecholamine release, triggering intraoperative hypertensive crisis and increasing the risk for major morbidity [7].

Improvement of operative and anesthetic techniques, such as laparoscopy and new anesthetic drugs, and preoperative medical preparation, has decreased the risk of perioperative hemodynamic instability. The perioperative management includes alpha-receptor blockade, suitable drugs for anesthesia, volume expansion, and postoperative care [8,9]. Historically, the most critical factor that has drastically reduced these patients’ perioperative morbidity and mortality is meticulous preoperative preparation. However, no clear guidelines exist about the ideal drug for preoperative preparation, and the evidence from a randomized controlled clinical trial is unavailable [10,11]. The optimal alpha-blockade strategy achieves a seated blood pressure (BP) < 130/80 mmHg, withstanding systolic BP > 90 mmHg. Traditionally, the long-acting, irreversible, non-selective alpha-blocker phenoxybenzamine is initiated 7–14 days preoperatively, and patients present side effects such as nasal congestion, preoperative orthostatic hypotension, and postoperative hypotension [11]. Short-acting, selective alpha-blockers, as doxazosin, have been promoted as alternatives, and they are associated with less preoperative orthostatic hypotension and shorter postoperative hypotension duration than phenoxybenzamine [10]. However, competitive inhibition can be overcome by high levels of catecholamines, and the antihypertensive effect of selective alpha-blockade may not be as potent [9].

Despite the preoperative preparation, 27.3% of the patients experienced hemodynamic instability intraoperatively, which must be promptly and carefully managed intraoperatively [12]. Short-acting drugs with an established safety profile (sodium nitroprusside, nitroglycerine, magnesium, and esmolol) permit good intraoperative blood pressure and heart rate management. The most common complication after tumor removal is hypotension, which may require fluid therapy and vasopressor support. Postoperative management usually requires intensive care or high dependency unit admission [13]. Although the recommendation from many reports suggested that patients with hormonally functional PCC should receive proper adrenoceptor blockade [14], in one recent series, up to one-third did not receive pre-adrenalectomy alpha-blockade [15]. In a recent series, preoperative selective alpha-adrenoceptor antagonist had no benefit in maintaining intraoperative hemodynamic stability in patients with normotensive PCC.

Moreover, they increased the use of vasoactive drugs and colloid infusion compared to PCC, which received no alpha-blockers preoperatively. Whether normotensive PCC patients truly benefit from alpha-adrenergic antagonist preparation remains uncertain [16]. These data suggest that alpha-blockade may sometimes be deemed “unnecessary” in PCC associated with normotension/postural hypotension or apparently “non-functional”, not according to recent recommendations and society’s guidelines. We performed a multicentric retrospective case-control study of patients undergoing adrenalectomy for PCC from 2000 to 2017 at two University Hospitals.

## 2. Materials and Methods

We conducted a multicentric retrospective study at St. Orsola-Malpighi Hospital (Bologna) and AOUP “P.Giaccone” Hospital (Palermo). Patients undergoing laparoscopic adrenalectomy for pheochromocytoma from 2000 to 2017 were included in the study. All patients included in the study have a preoperative positive catecholamine test with high levels of plasma-free metanephrines or 24 h urinary fractionated metanephrine). We included both symptomatic and asymptomatic patients.

The first team performed preoperative blood pressure management with alfa-antagonist and beta-antagonist drugs, while the second team did not. Both institutions collected data of patients in a prospectively maintained adrenal database. We performed per-protocol analysis, including only patients with a final pathological diagnosis of PCC. Planned open and bilateral adrenalectomies were excluded from our analysis. Patients with missing anesthetic records were excluded. Patients were divided into two groups based on preoperative alpha-blockade strategy: patients in Group 1 were treated with alpha-blockade, non-selectively with phenoxybenzamine, or blocked selectively with doxazosin (*n* = 49), while patients in Group 2 did not receive alpha-blockade preoperatively (*n* = 14) but only miscellaneous, on-demand, antihypertensive drugs during the crisis. Preoperative data included patient age, sex, Body Mass Index (BMI), comorbidities such as heart vascular disease and hypertension, presence of typical symptoms of PCC (headaches, sweating, tachycardia, palpitation, and hypertensive crisis), dimension of the lesion at CT scan and duration of preoperative treatment in patients receiving alpha-blockade. In the group of patients treated preoperatively with alpha-blockers, both selective and non-selective were used. Patients treated with phenoxybenzamine (*n* = 37) started 2–7 days before hospital admission. Following admission, they received an additional 5–10 days of alpha-blockade with phenoxybenzamine in the hospital. A preoperative high-sodium diet and fluid intake were encouraged to reverse catecholamine-induced blood volume contraction. A beta-blocker was added in cases of tachycardia. The remaining patients were treated with doxazosin (*n* = 12). The starting dose of doxazosin was 1 milligram daily and was titrated in 1-milligram increments to the desired effect. Patients were considered adequately blocked when they achieved a blood pressure below 140/90 mmHg. Patients of Group 2 received daily blood pressure monitoring until the operation and continued their chronic cardiovascular therapy. All patients underwent a cardiac evaluation through a thorough history and physical examination; complete blood count, basic metabolic panel, electrocardiogram, and echocardiogram were performed in all patients. A radial artery line was routinely inserted in both groups to monitor intraoperative hemodynamics. Intraoperative systolic blood pressure (SBP) and diastolic blood pressure (DBP) were measured every 5 min. The anesthesiologists established access with at least two peripheral intravenous lines or the positioning of central access. Anesthesia methods were similar for both groups: propofol was used for induction, and vecuronium bromide thymopeptides was as a muscle relaxant, isoflurane and fentanyl were also used during anesthesia maintenance. Intraoperative data included SBP at induction of anesthesia and pneumoperitoneum. Furthermore, SBP and DBP were monitored at the end of the surgery. In all patients, laparoscopic adrenalectomy was performed, and no conversions occurred; in every hospital, all procedures were performed by the same surgeon with experience in laparoscopic adrenalectomy. No Intraoperative complications were recorded. All patients were treated by using hemostatic agents at the end of the operation. Anesthesiologists determined the thresholds for treating patient hemodynamics and decided whether to use fluid or vasoactive drugs (sodium nitroprusside, nitroglycerine and esmolol). We included in our variables the admission to the intensive care unit (ICU), the length of stay (LOS), postoperative complications, and mortality. Data, which were collected using Excel software, were analyzed by R Studio version 1.1.419 software. For synthesis and variability measurements of continuous values, we chose median and interquartile range (IQR). Univariate inference analysis was conducted using the non-parametric Wilcoxon test for non-normal distribution and the Student’s *t* test for the normal one as well as the Shapiro–Wilk test for continuous variables and Chi-square for proportion. Multivariate analysis was conducted using linear regression models. The values were considered significant when *p*-value < 0.05.

## 3. Results

Demographics and preoperative variables are presented in Table 1.

The age and sex were comparable, whereas a trend was seen for a more significant proportion of women in both groups. Overall, the median age was 56 years in Group 1 and 57 years in Group 2, while 53% and 71.4% were women, respectively, and no statistical differences were detected in the two groups. The BMI of the patients in Group 2 was significantly greater (median 27, IQR 24.00–28.00, and median 24, IQR 21.25–26.75, *p*-value 0.0006).

There was no statistical difference in comorbidity, such as heart vascular disease and hypertension, in the two groups, even if many patients in Group 1 had preoperative major cardiovascular disease.

Dimension of the lesion detected at the CT scan was larger in patients of Group 2 (median 4.5 cm, IQR 3.63–5.88, median 3.2, IQR 2.38–8.70 respectively, *p*-value 0.0008), even if in Group 1, laparoscopic adrenalectomy was also performed for lesions larger than 6 cm. All patients in Group 1 were treated using alpha-blockers preoperatively, while only 28 patients (57.14%) needed beta-blockers during preparation. Intraoperative and postoperative results are summarized in Table 2.

Major postoperative complications were observed in 8.5% (*n* = 5) of patients in Group 1, while no major complications were detected in Group 2 (*n* = 0, *p*-value 0.49). The complications were two cases of pneumonia, two postoperative fluid collections in the surgical site, and one postoperative hematoma in the trocar site.

Moreover, no mortality was reported in either group. The use of advanced hemostatic agents was observed in 44.9% (*n* = 22) of patients in Group 1 and 100% (*n* = 14) of patients in Group 2 (*p*-value < 0.001). No Group 2 patients were admitted to the ICU, while 73.5% of Bologna patients were transferred to the ICU, resulting in an increase of one day in the median of the length of stay (*p*-value 0.026). Patients in Group 2 were readmitted to the ward after a few hours of observation and semi-intensive hemodynamic monitoring in the operating complex. During hospitalization in the ward, all patients of both groups were monitored hemodynamically with non-invasive methods. At the induction of anesthesia, SBP was 130 (IQR 120–150) mmHg in Group 1, while in Group 2, a lower SBP (median 115 mmHg, IQR 110–125, *p*-value 0.001) was detected. Instead, during the induction of pneumoperitoneum, SBP was 120.00 (IQR 100.0–128.8) mmHg for Group 1 and 110 (IQR 110.0–120.0) mmHg for Group 2, with no statistical difference (*p*-value 0.3570). At the end of the surgery, patients in group 1 had an SBP of about 110.0 (IQR 100.0–120.0) mmHg, while in Group 2, this was about 117.5 (110.0–120.0) mmHg. This difference has a low significance with a *p*-value of about 0.051. Moreover, DBP at the end of surgery was similar in both groups, with no statistical difference at univariate analysis. We analyzed the difference in SBP at induction and DBP at the end of surgery through multivariate analysis. The pre-surgery treatment was the only almost statistically significant variable at the multivariate analysis of DBP at the end of surgery (Table 3).

## 4. Discussion

Adrenalectomy for pheochromocytoma is reported with mortality close to zero in recent studies. The dogma of preoperative fluid and hypotensive drug administrations is widely applied in patients scheduled for pheochromocytoma removal and is assumed to benefit operative outcomes. This paradigm is only based on historical studies of non-standardized practices and criteria for efficacy, with no control group [1,12,15]. With advancements in surgical and anesthetic techniques, severe morbidity and mortality associated with the surgery are low in high-volume centers [2]. The dogma of preoperative blood pressure management is assumed to have a beneficial effect on operative outcomes, but this paradigm is only based on historical studies of non-standardized practices. Recent improvements in anesthetic management could permit an intraoperative reasonable blood pressure control without preoperative treatment, reducing postoperative hypotension. Better knowledge of the disease, efficiency of available intravenous short-acting vasoactive drugs, and careful intraoperative handling of the tumor make it possible to omit preoperative preparation in most patients scheduled for pheochromocytoma removal. Ulchaker et al. [17] reported that 30% of patients received no medication 24 h before surgery. Intraoperative mean blood pressure levels were similar in patients who were not treated preoperatively with antihypertensive medications [17]. Boutros et al. compared 31 patients receiving alpha-blockade preparation with 29 patients who did not receive any hypotensive drugs, and no difference in perioperative mortality or morbidity was found. However, intraoperative blood pressure rise was a little higher in the untreated group [18]. Shao et al. compared pheochromocytoma receiving preparation with doxazosin with a group of patients who did not receive any medication [16]. The intraoperative blood pressure and heart rate were similar in the two groups, whereas the intraoperative colloid transfusion was significantly greater in patients receiving doxazosin. DBP intraoperatively tended to be higher in patients without preoperative a-blockade but was not significant [16]. No severe hypertension/hypotension or tachycardia/bradycardia was detected during surgery in both groups. However, a1-blockade preparation increased the use of vasoactive drugs and intraoperative colloid fluid to maintain their blood pressure stability [16].

Furthermore, the use of noncompetitive a-adrenergic antagonist phenoxybenzamine leads to longer-lasting intraoperative hypotension that requires greater use of vasopressors [19]. Our series preoperative administration of a-adrenergic blockade did not improve the SBP during the operation compared to cases without preoperative antihypertensive management. No severe cardiovascular complications were detected during surgery in both groups. Increased knowledge of the disease, continuous arterial pressure monitoring, fast-acting vasoactive agents, and improvement of the surgical approach have dramatically improved the outcome of patients undergoing adrenalectomy for pheochromocytoma [8,19]. The anesthetic possibilities have transformed, and perhaps the time is ripe to change the management of these patients reducing waiting times for surgery and the risk of postoperative hypotension. Routine preoperative administration of fluids and hypotensive drugs is not supported by any evidence-based study [11]. There are only a few studies on this topic in literature due to the rarity of this disorder. A randomized prospective trial with a greater number of cases is required to confirm whether the a-adrenergic blockade is necessary or not as preoperative management for pheochromocytoma. This study has some limitations: first, the sample size for the group without preoperative treatment was small; second, the bicentric design could introduce some bias producing significant differences in the results, such as those in the use of hemostatic agents or postoperative intensive care; third, the difference in BMI and the size of the tumor could impact the results.

## 5. Conclusions

In conclusion, this is a small retrospective study and does not attempt, in any way, to influence the perioperative management habits of patients undergoing pheochromocytoma adrenalectomy surgery. In our experience, careful surgical handling of tumor tissue during laparoscopic resection, limited intraabdominal pressure, adequate depth of anesthesia and muscular relaxation, and fast-acting vasoactive agents are the only proven means to avoid intraoperative hypertension. Preoperative strict blood pressure control is possibly a dogma today.

## Figures and Tables

**Table 1:** Basal characteristics of the two groups.

Variable	Preoprative Anti-Hypertensive Management (n = 49)	Not Preoperative Anti-Hypertensive Management (n = 14)	*p*-Value
Age (years)	56.00 (IQR 49.00–70.25)	57.00 (IQR 44.00–70.00)	0.5627
Female sex	26 (53.06%)	10 (71.4%)	0.3583
BMI (kg/m^2^)	24.00 (IQR 21.25–26.75)	27.00 (IQR 24.00–28.00)	0.0006
Heart disease	11 (22.45%)	0 (0.00%)	0.1147
Hypertension	8 (16.33%)	3 (21.43%)	0.3288
Symptomatic	28 (57.14%)	10 (71.43%)	0.5132
Alpha-blocker	49 (100.00%)		
Beta-blocker	28 (57.14%)		
Calcium channel blocker	2 (4.08%)		
CT-dimension (cm max)	3.2 (IQR 2.38–8.70)	4.5 (IQR 3.63–5.88)	0.0008
Duration of preoperative treatment (days)	10.00 (8.00–18.00)		

BMI: Body Mass Index; IQR: interquartile range.

**Table 2:** Results of univariate analysis.

Variables	Preoprative Anti-Hypertensive Management (*n* = 49)	Not Preoperative Anti-Hypertensive Management (*n* = 14)	*p*-Value
Major complications	5 (10.20%)	0 (0.00%)	0.4933
Need for advance intra-operative haemostasis	22 (44.89%)	14 (100.00%)	<0.001
ICU admission	36 (73.47%)	0 (0.00%)	<0.001
Length of stay (days)	5 (IQR 4–6)	4 (IQR 4–4)	0.0260
SBP induction (mmHg)	130 (IQR 120–150)	115 (IQR 110–125)	<0.001
SBP pneumoperitoneum (mmHg)	120 (IQR 100–128.8)	110 (IQR 110–120)	0.3570
SBP end surgery (mmHg)	110 (IQR 100–120)	117.5 (IQR 110–120)	0.0513
DBP end surgery (mmHg)	60 (IQR 50–70)	60 (IQR 60–63.75)	0.6291

ICU: intensive care unit; SBP: systolic blood pressure; DBP: diastolic blood pressure.

**Table 3:** Results for difference in diastolic blood pressure at end of surgery (multivariare analysis).

Variables	Beta	*p*-Value
Pre-surgery treatment	9.74801	0.058068
Age	0.01569	0.901548
Sex female	0.97157	0.742060
BMI	−0.22330	0.651397
Hypertension	−2.72738	0.432634
Heart Didease	−3.62446	0.363887
Diabete	−5.99645	0.153887
ASA score	0.39027	0.901152
Symptoms	−4.11863	0.291716
Incidentaloma	−1.28322	0.754422
CT Dimension	0.7616	0.846408

BMI: Body Mass Index ASA: American Society of Anesthesiologists.

## Data Availability

The data can be requested by contacting the corresponding author.
